# Exploring clonality of *Mannheimia haemolytica* in beef cattle

**DOI:** 10.1128/spectrum.00278-26

**Published:** 2026-04-27

**Authors:** Nicholas D. Hacker, Matthew A. Scott, Lee J. Pinnell, Enrique Doster, William B. Crosby, Amelia R. Woolums, Cassidy L. Klima, Paul S. Morley, Robert Valeris-Chacin

**Affiliations:** 1 Department of Veterinary Pathobiology, College of Veterinary Medicine and Biomedical Sciences, Texas A&M University 14736, Canyon, Texas, USA; 2 Department of Large Animal Clinical Sciences, College of Veterinary Medicine and Biomedical Sciences, Texas A&M University 14736, Canyon, Texas, USA; 3 Department of Pathobiology and Population Medicine, College of Veterinary Medicine, Mississippi State University 5547, Starkville, Mississippi, USA; University of Georgia College of Veterinary Medicine 70734, Athens, Georgia, USA

**Keywords:** *Mannheimia haemolytica*, beef cattle, BRD, clonality, WGS, nanopore

## Abstract

*Mannheimia haemolytica* is a pathobiont bacterium of the upper respiratory tract in cattle and is associated with acute bovine respiratory disease (BRD). Despite research into bacterial pathogens and their associations with BRD, little is known about how bacterial variant dynamics interplay with disease progression. Therefore, our objective was to explore the clonal diversity of *M. haemolytica* in beef cattle utilizing whole genome sequencing. Ninety-nine *M. haemolytica* isolates from a commingled cohort of 33 cattle sampled in a prior study were selected for analysis. Long-read whole-genome sequencing was conducted to evaluate antimicrobial resistance genes, virulence factors, plasmids, and single nucleotide polymorphisms. Clonal lineages were derived using multi-locus sequence typing (MLST), a recently developed genomic sequence variant (GSV) method, and a core SNP-based clustering analysis with the average linkage method. We identified no differences in pathogenic profiles across isolates. We identified a single sequence type (ST1) and a single GSV (GSV 2) utilizing the MLST and GSV methods, respectively. The core SNP-based average linkage clustering analysis produced different clonal lineage estimations depending on the core SNP cutoff values (2 to 18 clusters). All estimations were greater than the single sequence type and GSV suggested by the MLST and the GSV methods. BRD status did not correlate with clonal interpretations. Our results suggest that method selection can influence the clonal interpretation of *M. haemolytica* within a population but may not reflect pathogenic capability. Alternate methods that increase discriminatory power or transcriptional profiles may provide new insights and should be evaluated.

**IMPORTANCE:**

Bovine respiratory disease remains a major challenge for cattle health and production, yet the population dynamics of key bacterial pathogens remain poorly understood. Using long-read whole-genome sequencing, this study shows that clonal diversity estimates of *Mannheimia haemolytica* are highly dependent on the analytical method employed, despite uniform virulence gene content. These findings highlight the importance of method selection in genomic epidemiology and caution against overinterpreting clonal structure as a proxy for pathogenic potential.

## INTRODUCTION

*Mannheimia haemolytica* is a common pathobiont bacterium of the upper respiratory tract in feedlot cattle (1). This species is associated with bovine respiratory disease (BRD), a multifactorial disease with immense economic burden on the cattle industry (2–4). Despite continued research into BRD pathogens and the determinants for disease, BRD remains a major burden on animal welfare and industry profitability (5). The signals that trigger *M. haemolytica* to shift from a commensal to a pathogenic bacterium remain unclear. Previous research points to cold stress, transportation stress, commingling, and viral infection of cattle as likely events that allow for bacterial colonization of the lower respiratory tract (6–11).

Several genetic determinants of *M. haemolytica* have been identified that influence pathogenicity (12–14). Virulence genes that are carried by *M. haemolytica* include *lkt*, *adhs*, *gs60*, *gcp*, *tbpB*, and *nmaA*, which facilitate the evasion of host defenses and colonization of the lower respiratory tract in cattle (12–14). Antimicrobial use in the feedlot industry for management of BRD has been tied to the increase of antimicrobial resistance genes (ARGs) in etiologically important bacteria (15). Common antimicrobial classes used for management of *M. haemolytica* infections are fluoroquinolones, macrolides, tetracycline, and amphenicols (2, 13, 16, 17). The development of antimicrobial resistance to these drug classes in BRD pathogens limits the ability of veterinary practitioners to manage BRD (2, 13, 16, 17). The combined virulence and ARG profiles of *M. haemolytica* can vary widely across populations without clear association with disease, making classification of BRD-associated variants difficult to discern directly from pathogenic profiles (18). Identifying variants with reliable associations with disease would allow a deeper understanding of disease dynamics and the underlying genetic shifts in the corresponding bacterial populations (19).

Characterization of capsular surface antigens using the rapid agglutination plate test has been implemented to further parse variants within *M. haemolytica* populations. There have been 12 different serotypes proposed for *M. haemolytica* that have been studied for their associations with BRD (2, 20). Serotype A2 is often associated with healthy animals and is commonly isolated from the upper respiratory tract, while serotypes A1 and A6 are considered the most prominent serotypes associated with BRD and are isolated from the lower respiratory tract (1, 2). Although serotyping is implemented regularly in pathogenesis research, the resolution of the technique may not prove sufficient to understand variant dynamics (20–22). The drifting of capsular antigens has been observed within *M. haemolytica* following repeated subculture and raises concern about accurate variant classification utilizing this method (23).

Genomic characterization of bacterial isolates with pulse field gel electrophoresis (PFGE) and multi-locus sequence typing (MLST) methods is often used to increase reliability of classification (2, 24). Once the gold standard in laboratories, PFGE uses restriction enzymes to digest chromosomal DNA. The unique banding patterns that result can be compared as a genomic fingerprint for variant identification (25, 26). Multi-locus sequence typing methods utilize (usually) seven housekeeping genes for variant identification and can incorporate ARGs to increase discriminatory power compared to PFGE methods (27–29). Despite their ability to aid in variant identification, the discriminatory power of these techniques can vary depending on species-specific characteristics and method optimization (27). To overcome the shortfalls of PFGE and MLST methods, pathogen surveillance systems have shifted to whole-genome sequencing (WGS) methods (30, 31). The specific technique utilized for WGS can vary; however, all methods aim to fully sequence the entire genome of a single organism. For bacterial pathogens, this is most commonly a singular isolate or culture.

Variant identification is reliant on clonal interpretation, yet clonality remains elusive to define (32). Classification of variants is complicated by mutation rates, recombination events, and selective pressures within and between animal hosts (32, 33). Furthermore, gene mobility may not align with ecological or geographical preconceptions, making variant dynamics difficult to study with multi-clonal systems (34). WGS has proven effective in distinguishing clonal lineages in several instances, improving the ability to study transmission dynamics (33, 35). Therefore, the objective of this study was to explore the clonal diversity of *M. haemolytica* in beef cattle utilizing whole-genome sequencing.

## MATERIALS AND METHODS

### Selection of *Mannheimia haemolytica* isolates

The *M. haemolytica* isolates were selected from a prior study evaluating swab type and *M. haemolytica* detection in the upper respiratory tract of cattle (36). In brief, two groups of 60 animals each were sampled using a single nasal swab and two nasopharyngeal swabs (double-guarded and proctology swabs) for *M. haemolytica* isolation. Animals were housed in six pens (10 animals each) and sampled 14 days after arrival at the West Texas A&M University Research Feedlot. For this study, *M. haemolytica* isolates were selected from 33 animals (all from the first group) for which all three swab types (nasal, double-guarded, and proctology) were positive, for a total of 99 *M. haemolytica* isolates. The selected *M. haemolytica* isolates were subcultured into Brain Heart Infusion (BHI) broth and incubated at 37°C and 5% CO_2_. After 24 h, *M. haemolytica* isolates were streaked on tryptic soy agar with 5% sheep blood and incubated at 37°C in 5% CO_2_. Plates were checked at 24 and 48 h for uniform growth of small, round, and pale colonies consistent with *M. haemolytica* (37). Additionally, the ATCC 33396 *M. haemolytica* strain was cultured and processed under the same conditions as the isolates recovered from cattle.

### DNA extraction

The *M. haemolytica*-inoculated BHI broths were used for DNA extraction using the DNeasy UltraClean Microbial Kit (Qiagen, Germany) on a QIAcube Connect device (Qiagen, Germany). Modifications were made to the protocol to maximize initial DNA yield. For this study, 2 mL of BHI broth was used for each sample in place of the 1.5 mL recommended by Qiagen to increase the post-centrifugation pellet size. Additionally, the bead-beating step was replaced with a heat block step at 65°C for 10 min to decrease DNA shearing, as recommended by the manufacturer (Qiagen, Germany).

### *Mannheimia haemolytica* qPCR

The molecular confirmation of the pure isolates as *M. haemolytica* was achieved via a simplex quantitative PCR reaction using SYBR Green chemistry. The *M. haemolytica*-specific primers used target the *lktD* gene (Forward Primer: CTGCAACAAAGCCGATATCTTT, Reverse Primer: TACGACTGCTGAAACCTTGAT) (38). Briefly, a final 20 μL solution containing 1× Quantabio Perfecta SYBR FastMix Low ROX buffer (Quantabio, Beverly, MA, USA), 500 nM of each primer, and 2 μL of DNA template was subjected to the following PCR program in a QuantStudio 3 real-time PCR system (Thermo Fisher Scientific, Waltham, MA, USA): a pre-cycling stage of 50°C for 2 min and 95°C for 5 min, followed by 40 cycles of 95°C for 15 s, 60°C for 45 s, and a melting curve stage of 95°C for 15 s, 60°C for 1 min, and a ramp to 95°C (held for 1 s). Cycle threshold (Ct) values <35 and a melting curve identical to that of the positive control (ATCC 33396 *M. haemolytica* strain) were required for identification of the isolates as *M. haemolytica*.

### Whole-genome amplification and library preparation

Prior to library preparation, the extracted DNA was amplified using the REPLI-g Midi Kit (Qiagen, Germany) following the manufacturer’s instructions. Amplified DNA was debranched to prevent pore clogging during sequencing using a T7 endonuclease enzyme, followed by a custom bead clean, as suggested by Oxford Nanopore Technologies (SQK-LSK112, Oxford Nanopore Technologies, Oxford, UK). Library preparation was performed using the Native Barcoding Kit 24 V14 (SQK-NBD114.24, Oxford Nanopore Technologies, Oxford, UK) following manufacturer’s instructions. *Mannheimia haemolytica* isolates with identical animal IDs were assigned to the same flow cell (*n* = 24 isolates per flow cell) to avoid batch effects when comparing swab types. Animal ID was randomized to flow cell, and unique barcodes were assigned to each *M. haemolytica* isolate within a given sequencing pool. DNA was sequenced on a PromethION P2 Solo or PromethION P2 Integrated sequencer (Oxford Nanopore Technologies, Oxford, UK) using the R10.4.1 flow cell and the Super Accurate Model (MinKNOW v23.07.15/Guppy v7.1.4 and MinKNOW v.24.11.8/Dorado v.7.6.7, respectively). Flow cells were run for 72 h. Isolates that failed to assemble at least one contig with over 1 Mb after sequencing were re-prepped and sequenced following the same procedures.

### Bioinformatics analysis

All bioinformatics analyses were performed using the Texas A&M High Performance Research Computing resources. Read quality was assessed for each sequence run using LongQC (version 1.2.0) with the ont-ligation flag (39). *De novo* assembly of reads was performed using Flye (version 2.9.4) with the nano-hq, read-error (set to 0.03), asm-coverage (set to 100), and genome-size (set to 2m) flags (40). Assemblies were polished using Medaka (version 1.12.0) with the resolve_mode used to determine the optimal model (r1041_e82_400bps_sup_v4.2.0) (41).

### Pangenome analysis

Genomes were annotated using Prokka (version 1.14.5) with the *M. haemolytica* ATCC 33396 reference genome (downloaded from the ATCC website) (42, 43). The pangenome was estimated using Roary (version 3.13.0) (44). Overall gene count differences from the *M. haemolytica* ATCC 33396 reference genome were computed using Python (version 3.11.13) (45). A heatmap of the pangenome was created using R (version 4.5.1) and pheatmap (version 1.0.13), displaying the presence or absence of genes in the core and accessory genome across all *M. haemolytica* isolates (46). A principal coordinate analysis (PCoA) plot using a Jaccard dissimilarity distance matrix containing the gene profiles of all *M. haemolytica* isolates was visualized using R (version 4.5.1) with the phyloseq package (version 3.22).

### Phylogenetic analysis

Genomes were assessed for single-nucleotide polymorphisms (SNPs) using Snippy (version 4.6.0) against the *M. haemolytica* ATCC 33396 reference genome (42, 47). Core SNP data were used to generate a maximum-likelihood phylogenetic tree using IQ-TREE (version 3.0.1) with the model finder and ascertainment bias correction options. A 10,000-iteration ultrafast bootstrap was used to obtain bootstrap estimates, with the minimum correlation coefficient for a UFBoot convergence set to 0.95 (48). The resulting phylogenetic tree was visualized in the interactive Tree of Life (iTOL version 7.0) (49).

### Clonality analysis

Clonality structure of the *M. haemolytica* isolates was evaluated using three approaches: MLST, a recently developed genomic sequence variant (GSV) method (50), and a clustering analysis with the average linkage method. For the MLST analysis, genomes were assessed for allelic matches using PubMLST against seven *M. haemolytica* housekeeping genes (*adk*, *aroE*, *deoD*, *gapDH*, *gnd*, *mdh*, and *zwf*) (29). Allelic combinations were used to determine the sequence type (ST) for all genomes using the PubMLST typing tool (51). For the GSV analysis, pseudoalignment against an index of *M. haemolytica* genomes classified into eight GSVs as described by Doster et al. (50) was performed. In brief, the raw reads classified as *M. haemolytica* using Centrifuge (version 1.0.4.2) were extracted for each isolate and pseudoaligned to the GSV index using Themisto (version 3.2.2) (52, 53). Relative abundance of the GSV groups was inferred using mSWEEP (version 2.2.1) (54). For the clustering analysis, an SNP distance matrix between all genomes using core SNP data were generated using snp-dists (version 0.8.2). Clonal clusters were identified using hierarchical clustering of the generated SNP distance matrix with the average linkage method across several SNP cutoff values (≤0, 2, 4, 8, 12, 16, and 20) (55). A bar plot was generated using Python (version 3.11.12) to visualize the resulting clone counts by the SNP cutoff values (45).

### Virulence and antimicrobial resistance genes

Genomes were assessed using Abricate (version 1.0.1) (56) and the following databases: VFDB (57), MEGARes (version 3.0) (58), ResFinder (59), PlasmidFinder (60), CARD (61), and NCBI AMRFinder (62) to determine the presence of ARGs (MEGARes, ResFinder, CARD, NCBI AMRFinder), virulence factors (VFDB), and plasmids (PlasmidFinder). Additionally, a manual BLAST of known ARGs and virulence factors associated with *M. haemolytica* was conducted using NCBI BLAST (version 2.16.0) (63) to identify alternate annotation names for ARGs and virulence factors not detected by Abricate.

### Statistical analysis

Associations between the pangenome output from Roary and metadata variables (swab type, animal ID, BRD status, and pen number) were evaluated using Scoary2 (version 0.0.15) with the phylogenetic tree generated in IQ-TREE (version 3.0.1) as a Newick file to adjust for population structure. A multiple testing flag was used with the Benjamini-Yekutieli correction (cutoff of 0.2) (48, 64). Associations between manually identified ARGs or virulence factors and metadata variables were also evaluated using Scoary2 (version 0.0.15) following the same settings.

## RESULTS

### Nanopore sequencing

Due to a power outage, the first set of *M. haemolytica* isolates (Mh_01–Mh_24) was only sequenced for 36 h. However, the coverage of reads did not differ significantly between the first set and the other sets sequenced for the full 72 h. Eight isolates (Mh_05, Mh_07, ATCC, Mh_24, Mh_31, Mh_36, Mh_38, Mh_46) were re-prepped following poor assembly: three from the first set, four from the second set, and one from the third set. The average DNA concentration after whole-genome amplification across all isolates was 208.61 ng/mL (SD = 101.76). An average Q score of twenty or higher was achieved by all isolates to a median read length of 12,000 bases. Six *M. haemolytica* isolates assembled into three contigs or less, while 94 isolates assembled into more than three contigs. The majority (*n* = 68) of *M. haemolytica* isolates contained at least one closed contig. The mean NG50 was 1,978,911.48 nucleotides and the LG50 was 1.15 contigs. The mean GC content across all assembled genomes was 41%.

### Pangenome analysis

**Fig 1:**
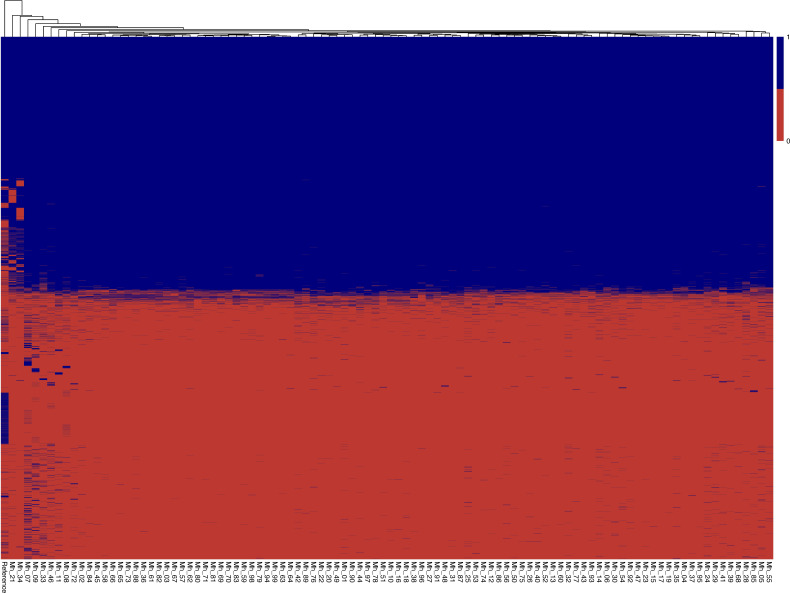
Pangenome heatmap of all identified genes with presence or absence in all *M. haemolytica* isolates, the ATCC 33396 isolate (Mh_ATCC), and the downloaded ATCC 33396 reference genome (Mh_Reference). Red positions signify absence of a gene, while blue positions signify presence of a gene.

**Fig 2:**
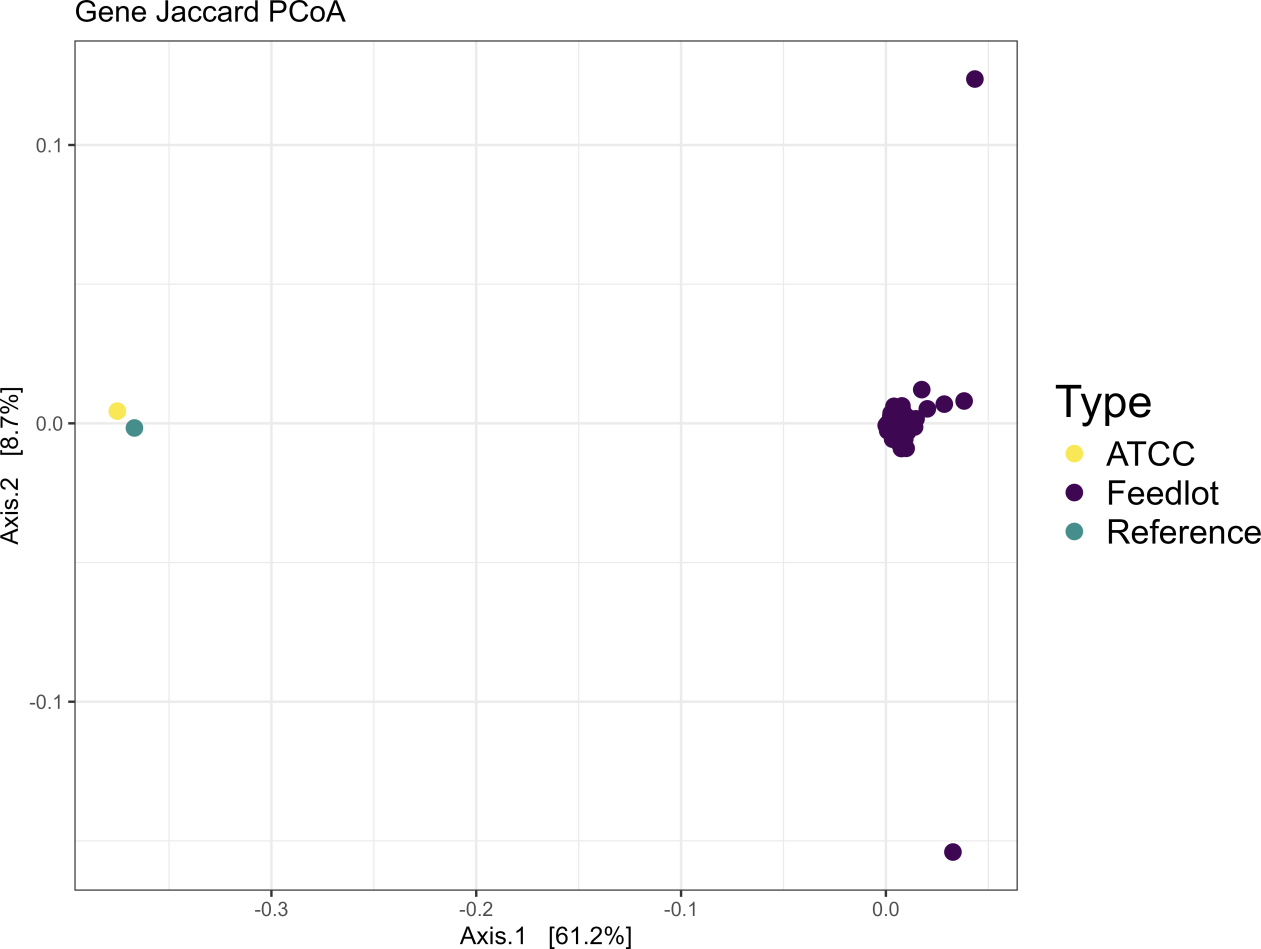
PCoA plot using Jaccard distances of gene presence or absence from all *M. haemolytica* isolates. The ATCC 33396 isolate and ATCC 33396 reference genome were added to the plot. The ATCC genomes are clustered separately from the rest of the *M. haemolytica* isolates in this study.

Analysis identified 5,499 genes in the pangenome of the *M. haemolytica* isolates. Of these, 2,407 genes were classified as core genes (present in 99%–100% of isolates), 222 classified as soft-core genes (present in 95%–99% of isolates), 169 classified as shell genes (present in 15%–95% of isolates), and 2,701 classified as cloud genes (present in 0%–15% of isolates). There were 66 identified mismatches in the gene presence/absence matrix between the ATCC isolate genome and the ATCC 33396 reference genome (downloaded from the ATCC website). A heatmap of gene presence/absence across the pangenome of all *M. haemolytica* isolates is visualized in Fig. 1. The PCoA plot of gene profiles (using the Jaccard dissimilarity distance matrix) showed clustering of the wild-type isolates into three distinct groups, separate from the ATCC genomes (Fig. 2).

### Phylogenetic analysis

**Fig 3:**
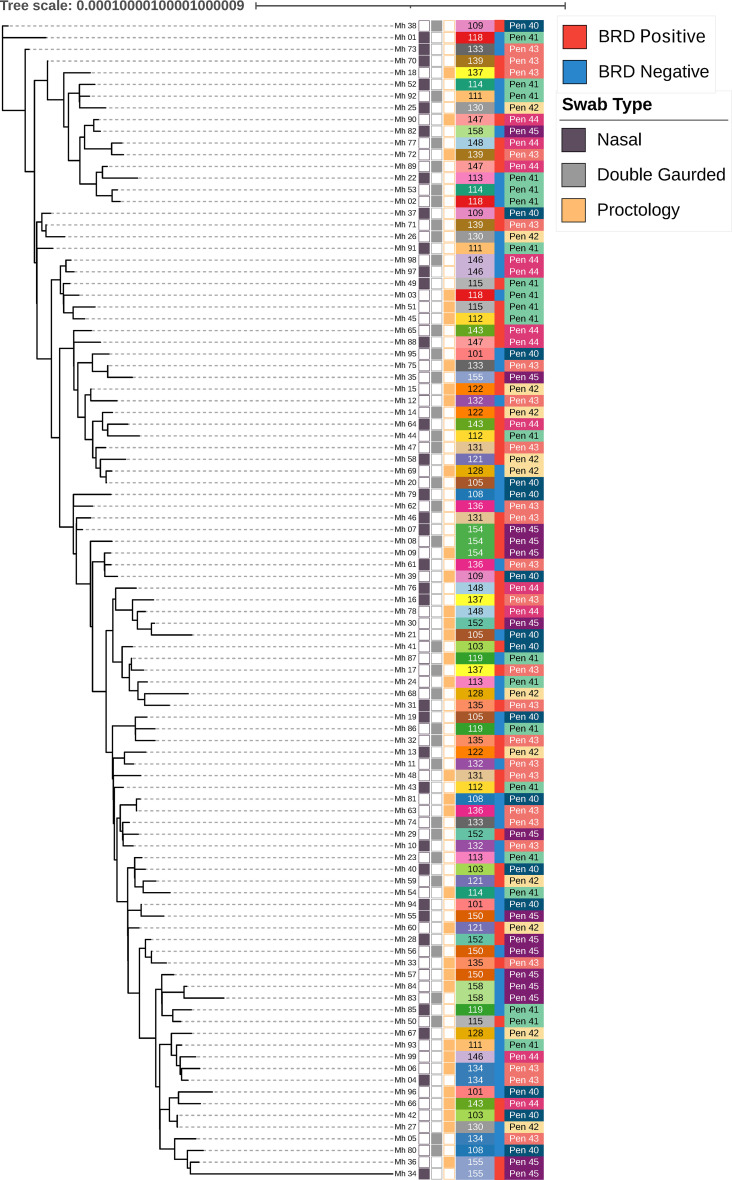
Core SNP-derived phylogenetic tree containing all isolates labeled with swab type, animal IDs, BRD status, and pen number. Nasal swabs are shown in purple, double-guarded swabs are shown in gray, and proctology swabs are shown in yellow. Animal ID numbers are presented with a unique color for all 33 animals. BRD-positive animals are shown in red and BRD-negative animals are shown in blue. The pen number is shown with a unique color for all five pens.

The ATCC 33396 reference genome was compared with the sequenced ATCC 33396 genome, and four total SNPs were identified across the genome (wgSNPs), likely due to sequencing and bioinformatic errors, as the two genomes were derived from the same clone. There was SNP variation identified between isolates and the ATCC reference genome (range: 8,754–10,161, median: 10,098). The core SNP phylogenetic tree showed no clear clustering of isolates with respect to metadata variables (Fig. 3).

### Clonal analysis

**Fig 4:**
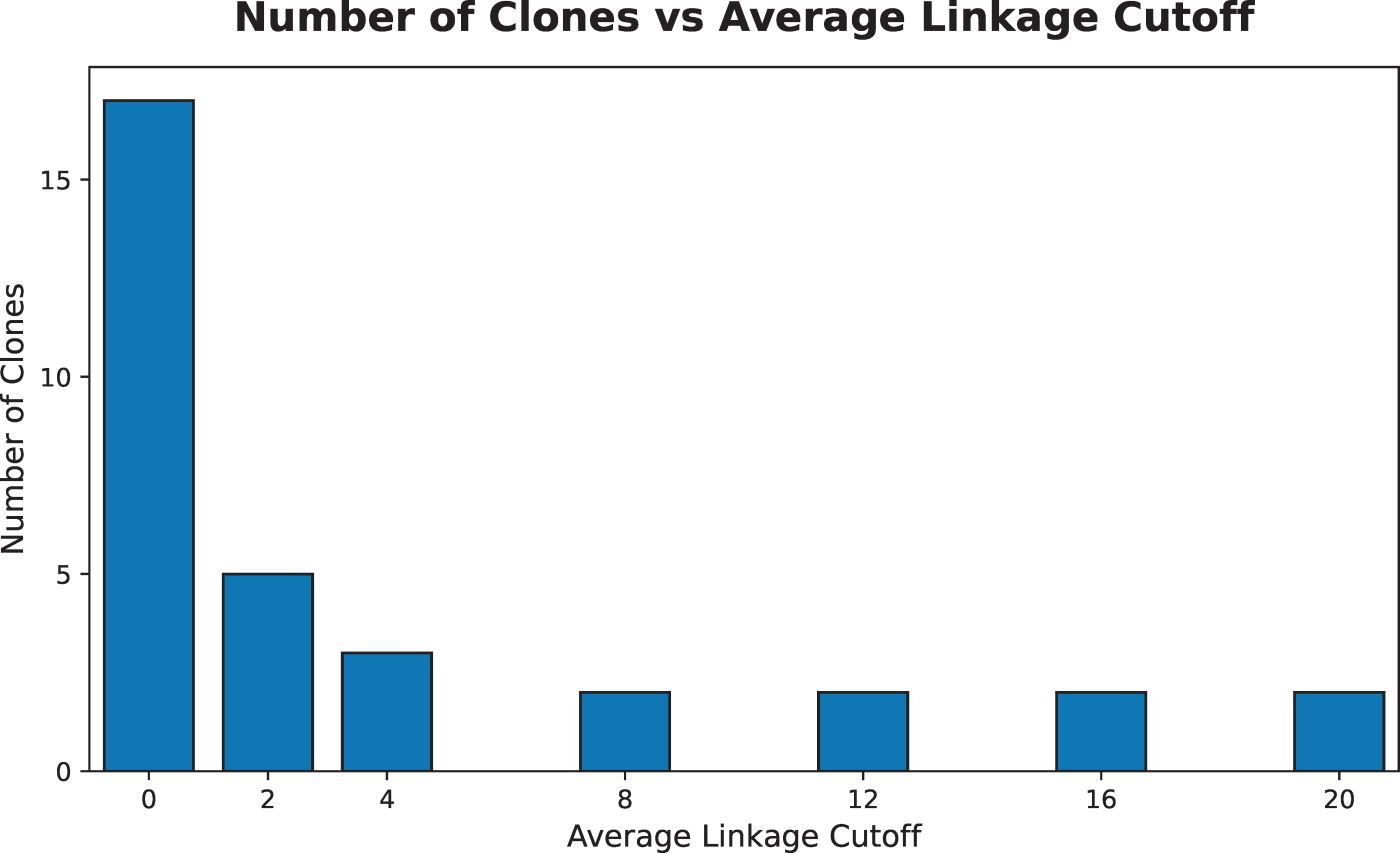
Bar plot of clone counts generated from the average linkage clustering analysis against different SNP cutoff values used in the grid search. A cutoff of zero SNPs produced the highest clone count, with 18 clones identified. Counts decrease as the cutoff value increases. Once an SNP cutoff value of 8 SNPs is reached, there are only two identified clones for the remaining cutoff values.

The assembled genomes matched to all seven housekeeping genes for *M. haemolytica* using PubMLST. All *M. haemolytica* isolates from this study, excluding the ATCC 33396 isolate, presented identical allelic combinations associated with ST1. The sequenced ATCC 33396 isolate and ATCC 33396 reference genome presented identical allelic combinations associated with ST2. All isolates were identified as the same GSV group (GSV 2). There were zero SNPs in the core genome between the ATCC 33396 reference genome and the ATCC 33396 isolate sequenced in this study. Depending on the core SNP cutoff used in the average linkage analysis to determine clonality (≤0, 2, 4, 8, 12, 16, 20), the wild-type *M. haemolytica* isolates were clustered in different groups. The most stringent cutoff value of zero core SNPs identified 18 unique clonal clusters. A cutoff value less than or equal to two core SNPs identified six unique clonal clusters, while a cutoff value of less than or equal to four core SNPs identified three unique clonal clusters. For the remaining cutoff values (≤8, 12, 16, 20), there were only two unique clusters identified, with the dominant cluster containing the majority (*n* = 98) of *M. haemolytica* isolates (Fig. 4). In all average linkage analyses, the ATCC 33396 isolate and ATCC 33396 reference were identified into their own unique clonal cluster.

### Virulence and antimicrobial resistance genes

Among all isolates, ARGs encoding resistance to aminoglycoside [*aph(3*″*)-lb, aph(3*″*)-la, aph(6)-ld*], macrolide [*erm(42)*], sulfonamide (*sul2*), and tetracycline [*tet*(H)] drug classes were identified. Additionally, the *gmhA/lpcA* virulence factors were identified in all isolates. Regions containing the following genes were identified in all isolates under alternative annotations using NCBI BLAST: *gs60, gcp, sodA, omp, ssa, lktCABD,* and *tbpB*. The complete list of ARGs and virulence genes, along with their known functions, can be found in Tables S1 and S2, respectively. No evidence of plasmids was obtained after running Abricate with PlasmidFinder.

### Statistical analysis

There was no variation in the ARGs and virulence factors identified with Abricate across metadata variables (swab type, animal ID, BRD status, and pen number), which precluded further statistical analysis.

## DISCUSSION

Using long-read sequencing, we successfully whole-genome sequenced 99 *M. haemolytica* isolates recovered from 33 animals using swabs to target the rostral nasal passage, the entire nasopharyngeal passage, and only the distal nasopharynx. Our results explored the potential diversity of clones using various methods within a population of *M. haemolytica*, while accounting for the variation in clonal lineage interpretations.

In this study, we evaluated the standard MLST method available for *M. haemolytica*, considered a gold standard for many years. The MLST scheme currently available for *M. haemolytica* encompasses seven housekeeping genes and cannot capture the full diversity of the organism across the genome. There is no proposed wgMLST or cgMLST scheme for *M. haemolytica,* limiting the current resolution of this technique. All isolates were identified as a single sequence type (ST1), suggesting a single clonal lineage in this study group. MLST can be used for variant analysis but offers limited resolution and discriminatory power. The use of WGS alongside MLST facilitates the direct identification of ARGs, virulence factors, and plasmids associated with pathogenesis from sequenced data. Pathogen surveillance systems have begun utilizing WGS methods as they offer high sensitivity, specificity, and ability to resolve clusters. For instance, PulseNet, the CDC’s national laboratory network for outbreak surveillance, began using WGS as the gold standard in its laboratories starting in 2019 (30). PulseNet prioritizes standardization across all participating laboratories (national and international) and favors cgMLST methods for comparability (31). However, the lack of verified schemes prevents the use of wgMLST or cgMLST methods for some pathogens, including *M. haemolytica*.

We analyzed the *M. haemolytica* isolates using a newly developed method for variant identification (50), which assigns isolates to GSVs. A GSV group represents a compilation of genomes evaluated using average nucleotide identity and is subject to structural changes based on the number of genomes included in the index and the number of GSV groups (50). All isolates were grouped into a single GSV, showing a similar trend to the MLST method with a single clonal lineage across the isolates. Our analysis was conducted using eight GSV groups, as using a higher number of GSVs has been shown to significantly decrease the accuracy of GSV classification (50).

Using the WGS data obtained in this study, a core SNP-based average linkage clustering analysis was conducted to leverage the higher discriminatory power of WGS methods compared to MLST. It has been suggested that between 0 and 20 SNPs within the core genome can be considered clonal (65, 66). Thus, a set of values encompassing this range was used for the purposes of this analysis. However, these SNP cutoff estimates are based on *Escherichia coli* and there is no obvious consensus for such cutoffs in *M. haemolytica* (65, 66). We observed that assuming complete clonality (monomorphic) within *M. haemolytica* isolates provided the highest count of clones with 18 clusters identified in the average linkage analysis. This is an unlikely scenario given mutation bias and mutation rates among bacterial species complicate evolutionary estimations (67). Recombination rates can differ between closely related lineages, suggesting that population structure may drive perceived clonality rather than valid phylogenetic interpretations. These recombination events are noted to be frequent and overwrite prior events across the same loci, further complicating clonal interpretations (68). Mutations and recombination rates play complex roles in bacterial phylogeny and are inextricable between closely related lineages, making it difficult to derive species-specific SNP cutoffs for clonality, let alone a universal cutoff applicable to all bacterial species. Beyond the biological limitations of deducing clonality, reference selection and differences in SNP-calling pipelines challenge the validity of interpretations made solely from cutoff values (69). When the cutoff for clonality is loosened, there is a large decrease in clonal clusters, as only six clusters are identified for a cutoff of less than or equal to two core SNPs. Furthermore, a cutoff of less than or equal to four core SNPs identified three clonal clusters. Once a cutoff of less than or equal to eight core SNPs was chosen, only two clonal clusters were detected, and this trend remained for the higher cutoff values used in the grid search. The variation in clonal interpretations suggests further refinement of the SNP cutoff used for *M. haemolytica* is needed to accurately report clonal lineages following this method.

The difference between the clonal clusters identified in the MLST and GSV analyses and those from the analysis with the core SNP cutoff values suggests that the latter achieves greater discriminatory power. When focusing on the core SNP-based average linkage clustering, there was variation in the identified clonal clusters between cutoff values lower than eight core SNPs. It is difficult to establish the true clonal diversity within these isolates without making further assumptions on which cutoff value to use. However, a maximum of 18 clones under the assumption of perfect clonality can be proposed for the isolates in this study. PFGE was not used in the clonality analysis, but it is considered less discriminatory than MLST methods. However, PFGE may provide an alternative insight (27). Of the three methods used in this study, the core SNP-based average linkage clustering was the most robust in discriminating clonal lineages without sacrificing granularity. The importance of method selection cannot be ignored when utilizing these techniques for variant classification, as it can alter downstream analyses and interpretations.

Understanding the diversity of clonal lineages in *M. haemolytica* populations can improve the classification of *M. haemolytica* variants and facilitate more robust source-tracking and transmission dynamics analysis (70), potentially parsing the underlying causes of variation in acute BRD cases within feedlot operations. One transmission dynamics study using PFGE suggested that *M. haemolytica*-associated BRD is not caused by a single virulent clone but by several clones within a commingled population (70). In another study, a decrease in *M. haemolytica* clonal diversity was observed at 13 days on feed via WGS (71). Furthermore, the potential dissemination of virulence genes by horizontal gene transfer within *M. haemolytica* populations highlights the implications of multiple clonal lineages interacting in the context of acute BRD within a commingled herd (72). Overall, there were no differences in pathogenic profiles among the clonal clusters identified by average linkage analysis, suggesting that clonal lineages may not influence *M. haemolytica* pathogenicity potential. However, this does not necessarily mean that clonal lineages will not influence the outcome of acute BRD in a herd. This topic warrants further research.

This study did not investigate the transcriptional profiles of the isolates; thus, we are unable to rule out the possibility that different clonal lineages may differ in expression of the pathogenic traits. Pathogenic profiles may not be fully representative, as the full scale of ARGs and virulence factors is most likely unknown. Database limitations are also a major concern, as common genetic determinants for pathogenesis within *M. haemolytica* were not recognized directly from Abricate. This is likely a compounding issue with poorly annotated genomes for this species. This study is also limited by the inherent nature of a core SNP average linkage clustering analysis. Reference selection can skew the initial SNP counts prior to core genome determination. Besides reference selection, addition or subtraction of genomes during the core genome analysis will alter the SNP distance matrix used for the average linkage clustering. The loss in comparability for SNP-based approaches can be seen as a disadvantage when contrasted with the highly standardized MLST methods. The reliance on culture-based methods for our WGS analysis may also underestimate the full clonal lineage diversity. Culture can also suffer from *M. haemolytica*’s phase variation and capsular drift properties, which impact serotyping (23, 73).

This study explored the heterogeneity in clonal interpretations that can be derived from current approaches using sequencing methods. The understanding of clonality in *M. haemolytica* frames the basis of accurate variant classification, and thus the validity of population dynamics based upon them. We consider that developing a *M. haemolytica* cgMLST/wgMLST scheme may provide a more comparable method for clonal interpretation, substantially improving on the discriminatory power of the current MLST scheme for *M. haemolytica*. The bottleneck to creating these schemes is sourcing high-quality genomes that capture the full diversity of *M. haemolytica* that can be encountered. Development of a more robust cutoff for SNPs in the core genome of *M. haemolytica* is also warranted. The observed variation between SNPs across the evaluated grid search in this study reiterates the importance of this metric on clustering. However, a similar issue to the wgMLST/cgMLST development is faced for SNP cutoffs as the capturing of the full diversity of *M. haemolytica* is needed to properly implement a viable cutoff. Future studies should also leverage metagenomic approaches and advanced NGS methods for sequencing, such as target enrichment and adaptive sampling, to overcome the limitations of culture-based methods. Metagenomic analysis may prove more robust in identifying the true scale of *M. haemolytica* clonal lineages present within and between the bovine hosts. However, there remain challenges with the generation of metagenomic-assembled genomes (MAGs) for reliable analysis. More robust methods for MAG generation are needed to fully evaluate the true diversity on a metagenomic scale.

### Conclusion

This study provides insight into the clonal diversity of *M. haemolytica* isolates from commingled commercial beef cattle and how method selection for variant classification can affect cluster interpretation. We identified a single sequence type (ST1) and a single GSV (GSV 2) utilizing the MLST and the GSV methods. The core SNP-based average linkage clustering analysis produced different clonal lineage estimations depending on the core SNP cutoff values, with greater discrimination than the MLST and GSV methods. However, further research is needed to determine which method is optimal for accurate classification of *M. haemolytica* variants. These findings underscore the importance of method selection for epidemiological studies and caution against relying on clonal structure as a proxy for pathogenic potential.

## Data Availability

De-identified data sets from this study are available in the SRA archives at the NCBI (https://www.ncbi.nlm.nih.gov/bioproject/PRJNA1374177/).

## SUPPLEMENTAL MATERIAL

Supplemental material spectrum.00278-26-s0001.docx Tables S1 and S2.

ASM does not own the copyrights to Supplemental Material that may be linked to, or accessed through, an article. The authors have granted ASM a non-exclusive, world-wide license to publish the Supplemental Material files. Please contact the corresponding author directly for reuse.
