# Supplementary material for: Exploring clonality of Mannheimia haemolytica in beef cattle
Source: Microbiol Spectr. 2026 Apr 27;14(6):e00278-26. doi: 10.1128/spectrum.00278-26 (PMC13228003; doi:10.1128/spectrum.00278-26)
Supplement: Supplemental material — Tables S1 and S2. [file spectrum.00278-26-s0001.docx]

Supplementary Materials

Table S1. Table of AMR genes identified in all isolates. Three aminoglycoside genes were identified, a single macrolide, sulfonamide, and tetracycline gene were also identified.

Table S2. Table of virulence genes identified in isolates. The VFDB database identified the *gmhA/IpcA* in all isolates. All other virulence genes were identified using NCBI blast.
